# The Best Strategy for the Black Hole Phenomenon between Intravascular Ultrasound and Optical Coherence Tomography

**DOI:** 10.3390/diagnostics14030281

**Published:** 2024-01-27

**Authors:** Cheng-Cheng Kan, Wei-Che Tsai, Cheng-Chung Cheng, Gwo-Ping Jong

**Affiliations:** 1 Department of Internal Medicine, Taichung Armed Forces General Hospital, Taichung 41152, Taiwan; engle31426@gmail.com; 2 Division of Cardiology, Department of Internal Medicine, Tri-Service General Hospital, National Defense Medical Center, Taipei 11490, Taiwan; wei_che7215@yahoo.com.tw (W.-C.T.); zephyr0204@gmail.com (C.-C.C.); 3 Department of Internal Medicine, Chung Shan Medical University Hospital, Chung Shan Medical University, Taichung 40201, Taiwan; 4 Institute of Medicine, Chung Shan Medical University, Taichung 40201, Taiwan

**Keywords:** black hole phenomenon, in-stent restenosis, intravascular ultrasound, optical coherence tomography

## Abstract

The black hole (BH) phenomenon is an intraluminal restenotic lesion. It was identified by intravascular ultrasound (IVUS) and optical coherence tomography (OCT) after intracoronary brachytherapy and drug-eluting stent implantation. Despite the similarity in the mode of action of brachytherapy and drug-eluting stent implantation, the BH phenomenon appears to be uncommon after drug-eluting stent implantation. Specifically, the BH phenomenon is better identified by OCT than by IVUS. Herein, we present a case of in-stent restenosis with suspected BH phenomenon on IVUS and confirmed by OCT.

In-stent restenosis (ISR) is a common complication that can occur following the placement of a coronary stent [1,2]. In recent years, the use of drug-eluting stents (DESs) has reduced ISR and major adverse cardiac events [3,4]. However, ISR after DES implantation still occurs [5,6]. The black hole (BH) phenomenon is when an intraluminal restenotic lesion image is obtained by intravascular ultrasound (IVUS) and optical coherence tomography (OCT) [7,8,9,10,11,12]. The development of ISR involves multiple factors, including biological, mechanical, and patient- and operator-related factors [13,14,15]. Intracoronary imaging is crucial for determining the specific mechanism of ISR, enabling tailored treatment strategies based on the identified cause. Therefore, we present a case of an ISR lesion with a suspected BH phenomenon on IVUS confirmed by OCT.

A 72-year-old man presented to our outpatient department with incremental exertional dyspnea and chest pain over the past 2 weeks. He had no history of cardiovascular risk factors, such as smoking and alcohol consumption, but had hypertension and type 2 diabetes (T2D). Additionally, he had a history of ischemic heart disease (chronic coronary syndrome) and underwent his first percutaneous coronary intervention (PCI) on 21 June 2022, which involved sirolimus-eluting stents (SESs) being placed at the proximal portion of the left anterior descending (LAD) artery and the proximal to the middle portion of the left circumflex artery. His ongoing medications included aspirin, clopidogrel, bisoprolol, atorvastatin, ezetimibe, furosemide, and hypoglycemic agents (pioglitazone, glimepiride, and dulaglutide).

During the cardiovascular outpatient department visit, his symptoms subsided, and ECGs displayed sinus rhythms without significant ST/T wave changes for 9 months. According to the history and positive findings of a treadmill exercise test, he was admitted for coronary angiography. On admission, his laboratory data revealed poor diabetic control (hemoglobin A1C of 8.3%) and controlled hyperlipidemia (total cholesterol, 132 mg/dL; triglycerides, 54 mg/dL; low-density lipoprotein, 55 mg/dL; and high-density lipoprotein, 59 mg/dL). Blood counts, serum electrolytes, and renal and liver function tests were within normal limits.

Coronary angiography on 27 April 2023 revealed severe ISR of the proximal LAD (Figure 1). A 60 MHz high-definition IVUS was performed, which revealed the homogenous echolucent appearance of ISR of the SES (Figure 2A) and a suspected BH phenomenon.

Given the significant constriction of the lumen area of the ISR lesion, obtaining a high-quality OCT image without balloon predilatation was challenging. Hence, we chose a 2.0 mm balloon to minimize the effect on the original lesion. OCT revealed a layered pattern with a superficial high signal-intensity band adjacent to the luminal surface and a signal-poor region near the stent (Figure 2B). According to the above imaging findings, the patient was diagnosed with a BH phenomenon through OCT.

A DES was implanted and subsequently post dilated using a noncompliant balloon. After PCI, the lumen surface appeared smooth, with no evidence of tissue prolapse or residual intraluminal thrombus, with a well-deployed stent. Thrombolysis in myocardial infarction III distal blood flow was achieved using angiography.

Following the placement of a coronary stent, ISR can commonly emerge as a complication [1,2]. Despite advances in technology, the occurrence rate of ISR has remained relatively consistent, affecting approximately 10% of patients after DES implantation [5,6].

The cause of ISR is multifactorial and can be attributed to various biological, mechanical, and procedural factors [13,14,15]. The mechanical factors primarily include stent expansion or fracture, whereas the biological factors involve localized inflammation leading to excessive neointimal growth and late neoatherosclerosis.

Previous epidemiological studies have revealed T2D as an important risk factor for developing ISR in patients following DES implantation [16,17]. Another study showed that the presence of T2D with elevated hemoglobin A1C levels is associated with ISR development in patients with DES [18]. The findings from this case highlight an increased ISR risk in patients with T2D and high hemoglobin A1C levels. Therefore, maintaining satisfactory sugar control in these patients may help prevent ISR following DES implantation.

The “BH” phenomenon, which has been characterized as an intraluminal restenotic lesion with a homogeneous black appearance (echolucent) on IVUS, was initially observed in patients following brachytherapy [7]. This phenomenon has also been identified in patients who have received SES implants [8].

DES improved BMS limitations but raised concerns about complications such as late restenosis and thrombosis. Late DES thrombosis is marked by delayed healing with impaired reendothelialization and persistent fibrin deposition. The exact cause of late restenosis remains unclear but may involve a delayed healing response to stent polymers and drugs.

Although the BH phenomenon is unusual in DES restenosis, establishing a universal mechanism is challenging. Tissue analysis of the BH revealed a primarily hypocellular matrix with areas rich in proteoglycans, possibly due to delayed vascular wound healing following SES implantation. The echolucent appearance was likely attributed to this hypocellular matrix and its high water content [9,10].

OCT revealed a layered structure with an inner layer with high signal intensity and outer layers with low signal intensity. The low OCT signal intensity was a result of restenotic tissue rich in proteoglycan and poor in collagen matrix or fibrin-rich thrombus formation. Although various restenotic tissues can exhibit low OCT signal intensity, the consistent border and structure in this case suggest that the BH phenomenon in OCT may have been due to the lack of organized mature connective tissue elements in the restenotic tissue [11,12].

Assessments by IVUS or OCT before DES restenosis treatment can help identify restenotic tissue characteristics and guide the development of an optimal strategy to prevent recurrent ISR. However, OCT is superior to IVUS for the diagnosis of the BH phenomenon after DES implantation.

We conclude that a BH phenomenon can occur after SES implantation. The described case suggests the superiority of OCT over IVUS rather than further research being necessary.

## Figures and Tables

**Figure 1:**
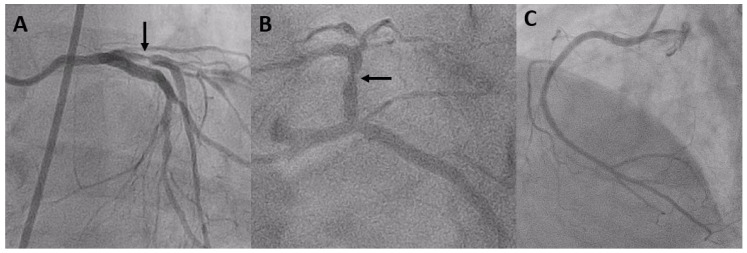
(**A**,**B**) Coronary angiography showing severe stenosis (black arrow) at the proximal portion of the LAD; (**C**) coronary angiography showing patent right coronary artery.

**Figure 2:**
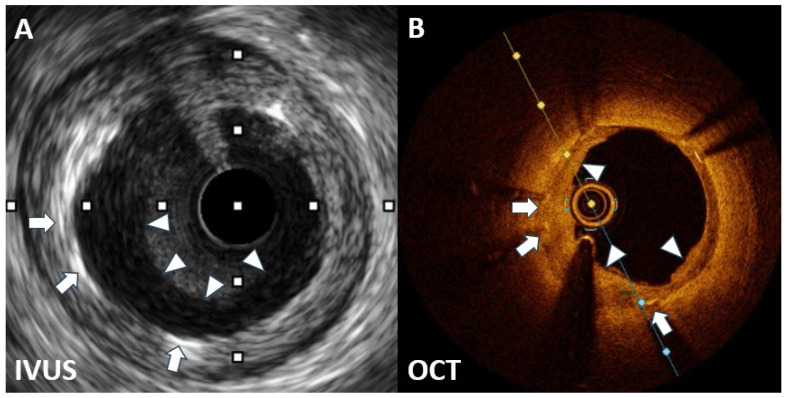
(**A**) Homogenous echolucent appearance of ISR of the sirolimus eluting stent. (**B**) A layered pattern with a superficial high signal-intensity band adjacent to the luminal surface, and a signal-poor region near the stent. (arrow: stent strut; arrow head: echolucent lesion in (**A**), and layered-pattern lesion in (**B**)).
